# Neural correlates of social and thematic semantics in autistic and non-autistic adults

**DOI:** 10.1093/scan/nsaf079

**Published:** 2025-07-24

**Authors:** Melissa Thye, Paul Hoffman, Daniel Mirman

**Affiliations:** School of Philosophy, Psychology & Language Sciences, University of Edinburgh, Edinburgh EH8 9JZ, United Kingdom; School of Philosophy, Psychology & Language Sciences, University of Edinburgh, Edinburgh EH8 9JZ, United Kingdom; School of Philosophy, Psychology & Language Sciences, University of Edinburgh, Edinburgh EH8 9JZ, United Kingdom

**Keywords:** semantic cognition, taxonomic, thematic, social, functional MRI

## Abstract

Conceptual knowledge—about objects, events, and social behaviour—is represented within the semantic system, but it is unclear if different conceptual categories engage the same portions of the system. This is perhaps most relevant for event-based, or thematic, knowledge and social knowledge which is acquired through social experiences. The present study investigated neural specialisation for social concepts by examining whether distinct semantic regions or hubs represent taxonomic versus thematic relations and social versus non-social relations. Specialisation was examined in two groups with different social experiences: autistic and non-autistic adults. There were minimal behavioural and no neural differences between groups, suggesting that differences in social experiences between autistic and non-autistic people may be better understood at the interactional level. In whole-brain analyses across both groups, taxonomic relations engaged the semantic control network to a greater extent than thematic relations did, and an overlapping portion of the rostroventral area of left angular gyrus was engaged by both thematic (relative to taxonomic) and social (relative to non-social) relations. Region of interest analyses revealed a more complex pattern within bilateral angular gyri. The results suggest that angular gyrus represents conceptual knowledge in a graded fashion, including specialisation for thematic and social relations.

## Introduction

The semantic system supports the representation of a wide-range of concrete and abstract conceptual knowledge, including knowledge about objects and their features, events, and social behaviour (McRae and Jones 2013). The breadth of this knowledge has motivated efforts to delineate if and where distinct and shared category representation exists within the semantic system. Central to this goal is characterising the extent to which the prevailing model of semantic cognition can account for the acquisition and representation of diverse forms of conceptual knowledge.

Extensive behavioural, neuroscience, and computational research has converged on a hub-and-spoke model of semantic cognition. This model describes a neurocomputational architecture in which the bilateral anterior temporal lobes (ATLs) serve as critical hubs integrating inputs from sensorimotor spokes that extend outside the hub (Lambon Ralph et al. 2017). This framework can explain patterns of general semantic impairment such as in semantic dementia as well as category-specific semantic deficits (Chen et al. 2017), but how it supports the representation of event-based or abstract knowledge is less clear.

To this end, there is evidence suggesting that the inferior parietal lobule (IPL), sometimes referred to as the temporo-parietal cortex, is a complementary semantic hub specialised for event-based relations (Mirman et al. 2017). Support for the dual-hub account stems from research examining the neural representation of feature-based, or taxonomic, relations compared to context-based, or thematic, relations in which both categories of concept engage the ATL but thematic relations especially engage IPL (Zhang et al. 2023, Zhang et al. 2024). The IPL is a large region, however, comprised of the supramarginal and angular gyri, which make distinct contributions to phonological (supramarginal gyrus) and semantic (angular gyrus) processing (Graves et al. 2023). In addition to a greater role in semantic processing, angular gyrus is functionally connected to the default mode network and, as a consequence, may have functional properties that particularly facilitate event or context-driven representation. It is consistently activated across wide-ranging tasks and is topographically situated at the convergence of several large neural networks, well-positioned to serve as a cross-modal hub (Seghier 2013, Igelström and Graziano 2017, Numssen et al. 2021, Humphreys and Tibon 2023). In addition, angular gyrus has a long temporal receptive window, integrating information over an extended timescale (Lerner et al. 2011).

Of note, however, is the considerable overlap between the neural systems that support semantic cognition and those that support social cognition, particularly in ATL and angular gyrus. A key point of intersection between these cognitive systems is in the representation of social concepts. Social concepts are a distinct category of semantic knowledge (Pexman et al. 2023) that emerge via experience as interpersonal agents in a highly social world (Borghi and Binkofski 2014, Borghi et al. 2018). As such, social concepts inherently involve diverse thematic associations. However, unlike object-based thematic relations, social associations often, but not always (Diveica et al. 2023), lack direct sensorimotor referents. Processing social concepts recruits portions of ATL, in particular the ventrolateral ATL which includes the anterior inferior and middle temporal gyri (Zahn et al. 2007, Olson et al. 2013, Binney et al. 2016), and this is true even for more abstract social processing such as mentalising (Balgova et al. 2022). Meta-analyses consistently implicate lateral ATL and portions of angular gyrus in social concept representation (Arioli et al. 2020, Zhang et al. 2021). Indeed, one of the most well-studied social cognition regions—temporo-parietal junction (TPJ)—overlaps with the IPL region implicated in thematic semantics. However, there is a fundamental difference in the hypothesized function of these regions for semantic and social cognition. In semantic cognition research, ATL and angular gyrus are described as cross-modal (or trans-modal) hubs that integrate conceptual information from different neural sources. In social cognition research, TPJ is typically considered a specialised module, possibly innately determined by evolutionary pressure from humans being social primates or via experience-dependent learning. In line with the latter account, it is possible that within angular gyrus, the semantic and social effects are at least partially dissociable, with social effects localised near middle temporal gyrus (MTG), anterior to the thematic effects (Lin et al. 2018, 2020). Alternatively, social and thematic relations may engage a shared portion of angular gyrus, consistent with the account that this area serves a domain-general role in semantic and social processing.

The present study examines neural specialisation for social concepts in light of two distinctions that are relevant for both semantic and social cognition. The first is comparing thematic versus taxonomic relations. There is substantial evidence that angular gyrus is more involved in processing thematic relations and mixed evidence regarding whether ATL is more important for taxonomic relations. To our knowledge, this is the first study to examine processing of thematic versus taxonomic relations alongside social versus non-social relations in order to investigate whether the same regions, or hubs, within the semantic system broadly support context-bound event representation. By including different kinds of content, we are able to test claims about specialisation of specific brain regions: ATL for taxonomic relations, angular gyrus for thematic and social relations. In addition, we are able to test the general hypothesis that social and thematic concepts rely on the same brain regions.

The second novel element in this study is examination of neural responses to social concepts among people with differences in social cognition, namely autistic and non-autistic adults. Like other abstract knowledge, social concepts are situated within context, being grounded in social experiences with other people (Barsalou 2003, Barsalou and Wiemer-Hastings 2005, Borghi 2022). Qualitatively different social experiences might drive differences in how the semantic system represents social information. Prior studies have reported differences in how autistic and non-autistic adults process general semantic information (Kamio et al. 2007) as well as social conceptual knowledge (Phan et al. 2021, Graves et al. 2022, Birba et al. 2023). However, studies examining social concept processing in autistic adults did not include comparisons of taxonomic and thematic relations, despite the fact that individuals display variability in preferential semantic processing style (Mirman and Graziano 2012) and these preferences remain stable across development (Dunham and Dunham 1995). Although largely unexplored, it has been suggested that autistic adults might have a taxonomic preference (Graves et al. 2022) which could subsequently impact how both thematic and social relations are processed. To address this question, the present study investigated both thematic and social relations in autistic and non-autistic adults. Contrary to early influential studies, recent work shows that autistic individuals do not have impaired social cognition relative to non-autistic individuals. Instead, the two neurotypes differ in how they process and communicate social and linguistic information (Crompton et al. 2020). As a result, we did not expect differences in task performance between autistic and non-autistic participants, but it is possible that there will be differences in neural response, suggesting differences in how social knowledge is represented and processed.

## Materials and Methods

### Participants

Participants were recruited through university and community networks and a local autism organisation. Recruitment was restricted to ensure that groups were matched on age and sex. A total of 55 participants took part in this study. Data from seven participants were excluded from analyses due to a reported history of neurological illness or dyslexia (*n* = 2), being a non-native English speaker (*n* = 1), a scanner malfunction (*n* = 2), or low accuracy on the task (*n* = 2), resulting in a final sample size of 48 participants (24 per group). These group sizes are comparable to other published fMRI studies. Post hoc sensitivity analyses indicated that the study had 80% power to detect effect sizes of Cohen’s *d* ≥ 0.82 with 24 participants per group (α  =  0.05) and *d* ≥ 0.41 for analyses conducted using the full sample (*N* = 48). All participants were classified as right-handed according to the Edinburgh Handedness Inventory (Oldfield 1971). Demographic information is provided in Table 1. All participants provided written informed consent and received £20 and a picture of their brain for taking part. Imaging was carried out at the Edinburgh Imaging Facility, RIE, at the University of Edinburgh. The study procedures were approved by the PPLS research ethics board at the University of Edinburgh. No part of the study procedures or analyses plans were pre-registered prior to the research being conducted.

**Table 1.** Participant demographics.

	Autistic	Non-Autistic	Comparison
	M (SD); range	M (SD); range	*P*-value
Age	27.46 (5.18); 19–35	27.00 (3.27); 19–32	.716
Vocabulary	37.92 (4.23); 24–47	39.65 (4.21); 30–47	.165
Matrix reasoning	20.96 (3.09); 9–25	21.79 (2.04); 17–25	.277
AQ-10	7.96 (1.60); 5–10	2.67 (2.16); 0–7	***<.001***
CATI total	173.38 (14.38); 145–202	107.75 (25.98); 54–144	***<.001***
RAADS-14	32.25 (7.04); 16–42	9.08 (7.20); 0–20	***<.001***
Sex (F: M)	15:9	15:9	
Gender (F:M:NB:NP)	8:6:6:4	14:9:1:0	
Language use (Mono: Multi)	23:1	18:6	.097
*Educational attainment*			***.007***
Less than bachelor’s degree^a^	12	4	
Bachelor’s degree	10	12	
Master’s degree	1	8	
Not provided	1	0	
*Race/ethnicity*			.240
White	22	21	
Mixed race	2	0	
Asian	0	2	
African	0	1	

M, mean; SD, standard deviation; AQ, autism quotient; CATI, Comprehensive Autistic Trait Inventory; RAADS, Ritvo Autism & Asperger Diagnostic Scale; F, female; M, male; NB, nonbinary; NP, not provided; Mono, monolingual; Multi, multilingual.

a All participants had completed secondary education. Group comparisons were done using two-sample *t*-tests for continuous variables and Fisher’s exact test for categorical variables. *P*-values below the standard significance threshold of .05 are italicized and bolded.

**Figure 1.**
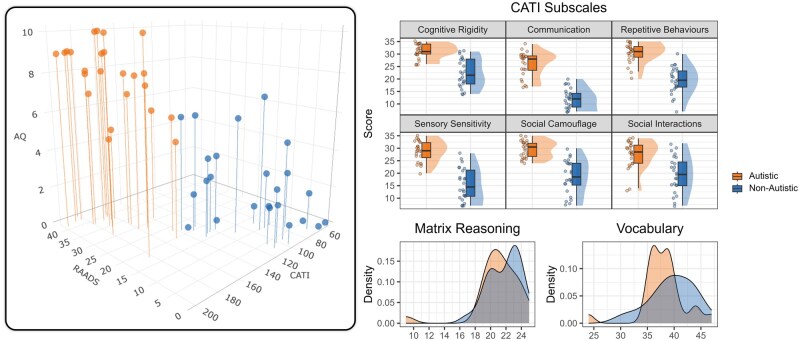
Scores on administered measures by group. The left panel is a three-dimensional scatterplot of AQ-10, RAADS-14, and total CATI scores showing where each participant (represented with a circle) scored on each measure relative to their scores on the other measures. Higher scores on all measures are indicative of an increase in autistic traits. The right panel shows the distribution of CATI subscales scores by group at the top and the matrix reasoning and vocabulary scores by group at the bottom. For the left panel and the top right panel, autistic participants are shown on the left and non-autistic participants are shown on the right. In both panels, autistic participants are shown in orange and non-autistic participants are shown in blue.

Participants completed an online eligibility survey, then a longer online intake survey (∼30–60 minutes) administered via Qualtrics once eligibility was confirmed. The intake survey included general demographic questions, the vocabulary and matrix reasoning items from the WASI-II, the AQ-10 (Allison et al. 2012), the 14-item version of the Ritvo Autism and Asperger Diagnostic Scale-Revised (RAADS-14; Eriksson et al. 2013), and the Comprehensive Autistic Trait Inventory (CATI; English et al. 2021). The items from the WASI-II vocabulary subtest (*n* = 28) were presented visually and orally using a standard British pronunciation. Given the medium of presentation, no time limit was imposed, participants were not asked follow-up or clarifying questions about their responses, and all items were administered. Compliance with the vocabulary and matrix reasoning items was monitored by tracking the number of times participant clicked out of the survey. This prompted a pop-up telling them to complete the survey in a single session without navigating to other webpages. Compliance was high across both groups, with the mean number of clicks off screen being less than 2. The two groups were matched on age, sex, vocabulary, and matrix reasoning, but were not matched on educational attainment with the non-autistic group, on average, having a greater proportion of advanced degrees than the autistic group. Although semantic knowledge increases with education and experience, we do not expect educational attainment to impact performance on the administered task given that the stimuli were familiar, high frequency words that were not intended to require advanced education to understand and were normed online with participants of varied educational backgrounds. The difference in educational attainment is more likely driven by differences in how the groups were recruited: non-autistic adults tended to be recruited through university networks whereas autistic participants were more often recruited through community networks. Autistic participants had either received a clinical diagnosis (*n* = 20) or self-diagnosed (*n* = 4), and the autistic group scored substantially higher on the AQ-10, RAADS-14, and CATI than the non-autistic group (Fig. 1).

### Materials

During the fMRI session, participants were presented visually with word pairs and asked to judge if they were semantically related or not. Each related word pair belonged to one of four conditions: taxonomic, thematic, social, and non-social. The details of the stimulus selection are provided in the supplemental materials.

The final set of stimuli included 220 trials, 60% of which (*n* = 132, *n* = 33 per condition) were critical trials and 40% of which (*n* = 88) were filler trials comprised of abstract (*n* = 44) or concrete (*n* = 44) words. Taxonomic and thematic word pairs were matched on number of letters, word frequency, concreteness, imageability, familiarity, semantic diversity, and cosine similarity, as were the social and non-social word pairs (Table 2). Grammatical class was matched within word pair and between taxonomic and thematic words (all nouns), but was more variable and unbalanced between social and non-social words (a mix of adjectives, verbs, and nouns). Grammatical class in English is somewhat flexible (e.g. many nouns can be used as verbs or adjectives/modifiers and vice versa, with the difference only evident in a sentence context), so when words are presented in isolation or in word pairs, their grammatical class is ambiguous. More generally, grammatical categories differ substantially in their semantic properties (e.g. nouns typically refer to objects while verbs typically refer to actions) and prior research suggests that it is these semantic properties, rather than grammatical class, that drive behavioural and neural differences in processing (Bedny et al. 2014). The stimuli are available Open Science Framework (OSF): https://osf.io/5xasw.

**Table 2.** Word pair properties across conditions.

Condition	^#^ Letters	Frequency	Familiarity	Concreteness	Imageability	Semantic diversity
Taxonomic	5.45 (1.76)	2.88 (0.59)	523 (58.28)	588 (33.41)	589 (38.27)	1.50 (0.20)
Thematic	5.23 (1.40)	2.95 (0.54)	536 (53.68)	585 (31.97)	581 (40.97)	1.52 (0.22)
Concrete filler	5.60 (1.53)	2.80 (0.56)	530 (62.16)	586 (33.79)	583 (35.41)	1.49 (0.24)
Social	6.83 (1.97)	2.60 (0.72)	528 (49.00)	326 (69.24)	406 (42.71)	1.80 (0.17)
Non-Social	6.39 (1.87)	2.57 (0.60)	513 (61.32)	341 (61.38)	390 (47.17)	1.82 (0.22)
Abstract filler	6.50 (1.52)	2.61 (0.73)	522 (41.57)	337 (50.95)	394 (50.24)	1.82 (0.17)

Mean values per condition with standard deviation in parentheses. Word properties were obtained from the English Lexicon Project (Balota et al. 2007).

# , number.

### Relatedness judgment task

At the start of the session, participants completed a practice task on a laptop outside the scanner. The first block of the practice task (*n* = 8 trials) was self-paced and feedback was provided after each trial. The final two blocks of the practice task (*n* = 16 trials each) were presented with the same timing as the experimental paradigm, but participants additionally received feedback on accuracy at the end of each block which included a listing of the related and unrelated word pairs. Where accuracy was low, the instructions were reviewed again alongside the word pairs. Responses were indicated via the laptop keyboard using the ‘z’ key for related and the ‘m’ key for unrelated. These keys were chosen because it allowed participants to practice the *left-related* and *right-unrelated* mapping used in the experimental paradigm.

**Figure 2.**
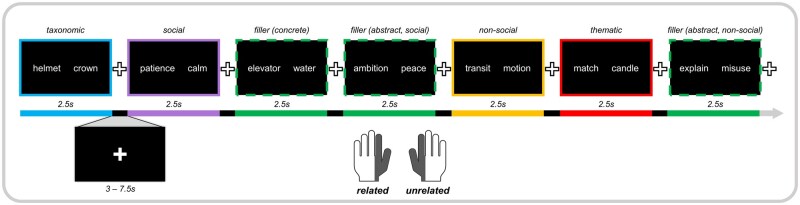
Semantic relatedness judgment task. Example stimuli are shown for taxonomic (blue), thematic (red), social (purple), and non-social (orange) trials. Unrelated, filler trials are shown outlined in dotted green lines. Filler trials included concrete and abstract social, and non-social words. Each trial was separated by a fixation cross with a jittered intertrial interval ranging from 3 to 7.5 s. On each trial, participants indicated whether word pairs were related (using left index finger or thumb) or unrelated (using right index finger or thumb).

During the scan session, participants completed two runs of the relatedness judgment task in which they were asked to judge the relations between taxonomic, thematic, social, non-social, or unrelated word pairs. Participants used hand held triggers to indicate if the words were related (left index finger or thumb) or unrelated (right index finger or thumb). Hand assignment was not counterbalanced across participants because we were only interested in analysing related trials and did not want differences in motor activation to drive spurious group or condition differences. All participants were right handed, so hand preference was approximately the same across participants. The runs were counterbalanced across participants and trials within the runs were randomised. All word pairs were presented to participants without repetition. Each trial was presented for 2.5 s followed by a jittered inter-trial interval in which a fixation cross was presented for 3 to 7.5 s (*M* ≈ 5 s). A schematic of the task and timing is presented in Fig. 2. The practice and experimental task were programmed in PsychoPy version 2021.2.1 (Peirce et al. 2019).

### Image acquisition

Images were acquired on a 3 T Siemens Prisma scanner with a 32-channel head coil, with the exception of 2 participants for which a 64-channel head coil was used due to a malfunction with the 32-channel coil. A high-resolution T1-weighted structural image was acquired for each participant using an MP-RAGE sequence with 0.8 mm isotropic voxels, TR = 2.62 s, TE = 4.49 ms. A multi-echo EPI sequence was used to acquire functional images and included 46 slices covering the whole brain with echo time (TE) at 13, 30, and 48 ms, repetition time (TR) = 1.7 s, flip angle = 73°, 80 × 80 matrix, field of view = 240 mm, reconstructed in-plane resolution = 3 mm × 3 mm, slice thickness = 3 mm (no slice gap) and multiband factor = 2. A multi-echo acquisition protocol was used to minimize signal drop out in the ventral temporal regions. The relatedness judgment task was presented across two 887.2 s runs (516 volumes) after which participants completed a run of movie-viewing which is not analysed in the present study.

### Behavioural analysis

Filler trials were removed prior to analysis. Participants were excluded from analysis if they missed more than two-thirds of the critical trials within a condition. A mixed effects logistic regression was used to model accuracy with a fixed group X trial type interaction term and a random effects structure consisting of by-subject random intercepts and slopes of trial type and a random effect of word pair. Analysis of participant reaction times was restricted to correctly answered trials and was carried out using a linear mixed effects model predicting reaction time with a fixed group X trial type interaction term and the same random effects structure as the accuracy model. Post-hoc pairwise comparisons between the regression coefficients of the conditions of interest were done using Wald tests.

### Whole-brain analyses

Images were pre-processed and analysed using SPM12 (https://www.fil.ion.ucl.ac.uk/spm/) and the TE-Dependent Analysis (tedana) Toolbox 0.0.7 (DuPre et al. 2021). Estimates of head motion were obtained using the first BOLD echo series. Slice-timing correction was carried out and images were then realigned using the previously obtained motion estimates. Tedana was used to combine the three-echo series into a single-time series and to divide the data into components classified as either BOLD-signal or noise-related based on their patterns of signal decay over increasing TEs (Kundu et al. 2017). Components classified as noise were discarded. After that, images were unwarped with a B0 fieldmap to correct for irregularities in the scanner’s magnetic field. Finally, functional images were spatially normalised to MNI space using SPM’s DARTEL tool (Ashburner 2007) and were smoothed with a kernel of 8 mm FWHM.

After removing filler and inaccurate trials, the events within the two runs were analysed using a single general linear model in AFNI (Cox 1996). For each condition, accurate trials were modelled as boxcar regressors with 2.5 s duration and convolved with a canonical hemodynamic response function. Trial-level reaction time was included as a parametric modulator in the analysis to control for variable response rates across subjects and trials. The six motion realignment parameters were included as nuisance regressors. The subject-level activation maps for each condition were used as inputs for a second-level group analysis using linear mixed-effects modelling with a fixed Group × Condition interaction term and random intercepts of subject using 3dLMEr in AFNI (Chen et al. 2013). Analyses were restricted to a grey matter mask generated by concatenating the subject-level grey matter masks. The condition (*Thematic* > *Taxonomic*, *Social* > *Non-Social*, *Abstract* > *Concrete*), group (*Autistic > Non-Autistic*), and group × condition simple effects were extracted from the model. Permutation-based cluster correction was implemented using 3dClustSim based on the spatial smoothness of the residuals file (Cox et al. 2017). This yielded a cluster size of 107 based on bisided first-nearest neighbour clustering and an uncorrected cluster forming threshold of *P* < .005 and an FWE-corrected threshold of *P* < .05. The resulting thresholds were applied to the group-level activation maps for each condition contrast. Results figures were generated using MRIcroGL (Rorden and Brett 2000). All analysis code, study materials, and results files are available on OSF: https://osf.io/5xasw.

### Region of interest analyses

**Figure 3.**
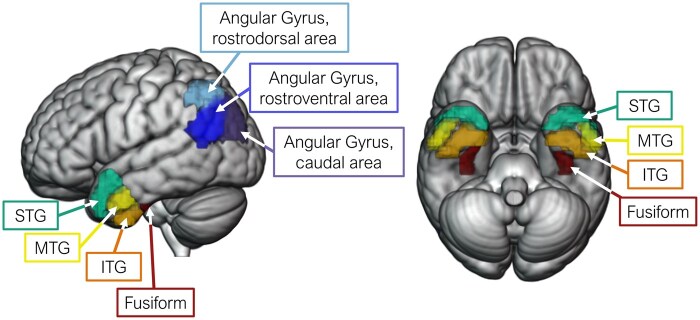
Regions of interest. Seven bilateral ROIs were used to capture engagement within ATL and angular gyrus. The left lateral surface is shown in the figure on the left and the inferior view is shown in the figure on the right.

To investigate the condition by group effects further, we conducted region of interest (ROI) analyses within ATL and angular gyrus, dividing each into subregions to examine graded contributions (Fig. 3). Bilateral ATL subregions were defined using the two most anterior segments from a prior study which parcellated the temporal gyri into anterior to posterior segments (Hoffman and Lambon Ralph 2018). They covered approximately the anterior one-third of each temporal gyrus. This resulted in four subregions: fusiform, inferior temporal gyrus (ITG), MTG, and superior temporal gyrus (STG). Bilateral angular gyri subregions were defined using the Brainnetome atlas (Fan et al. 2016) and consisted of a rostrodorsal area (A39rd), a rostroventral area (A39rv), and a caudal area (A39c).

The mean coefficient value within each of these ROIs were extracted from the subject-level statistical maps and used as the dependent variable in two mixed effects models. The first model tested the thematic and taxonomic effects across ROIs with fixed effects of group and the hemisphere × region × condition interaction. Random intercepts and random slopes for condition and hemisphere by subject were included to account for subject variability. The second model tested the social and non-social effects across ROIs using the same fixed and random effects structure. In both models, the group effect and all group two-way and three-way interactions were insignificant, so group interactions were dropped from the model. Model comparisons confirmed that including the group interaction term did not improve model fit.

## Results

### Behavioural results

**Figure 4.**
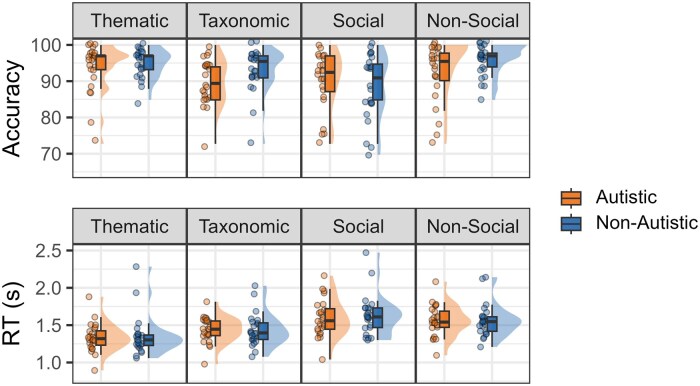
Accuracy and reaction time data by condition and group. RT, reaction time; s, seconds.

The behavioural results are provided in Table 3 and Fig. 4. There were no group differences in reaction time, and minimal group and condition differences in accuracy. Across groups, there were no significant differences in accuracy or reaction time between thematic and taxonomic conditions or between social and non-social conditions. The only statistically significant effects were as follows: (i) the non-autistic participants judged the taxonomic pairs more accurately than the autistic participants did (*Mdn_A_* = 89.40, *Mdn_NA_* = 95.50, *Est.* = −0.554, *SE* = 0.260, *P* = .033) and (ii) compared to the autistic group, the non-autistic group had significantly lower accuracy in judging social (*Mdn_A_* = 92.40, *Mdn_NA_* = 90.90) compared to non-social (*Mdn_A_* = 95.50, *Mdn_NA_* = 97.00) pairs (*Z* = 2.163, *P* = .031).

**Table 3.** Behavioural results.

	Accuracy			Reaction time		
Across groups	Estimate	SE	*P*	Estimate	SE	*P*
Thematic—taxonomic	0.773	0.361	.141	−0.094	0.037	.058
Social—non-social	−0.876	0.349	.058	0.052	0.036	.481
Autistic > non-autistic	Estimate	SE	*P*	Estimate	SE	*P*
Taxonomic	−0.554	0.260	***.033***	−0.001	0.056	.992
Thematic	−0.226	0.368	.539	−0.016	0.065	.812
Social	0.167	0.286	.559	−0.036	0.074	.624
Non-social	−0.642	0.368	.082	−0.005	0.060	.928
Autistic > non-autistic	*Z*		*P*	*Z*		*P*
Thematic—taxonomic	0.834		.404	−0.489		.625
Social—non-social	2.163		***.031***	−1.15		.250

*P*-values below the standard significance threshold of .05 are italicized and bolded. SE, standard error of the mean.

### Whole-brain results

**Figure 5.**
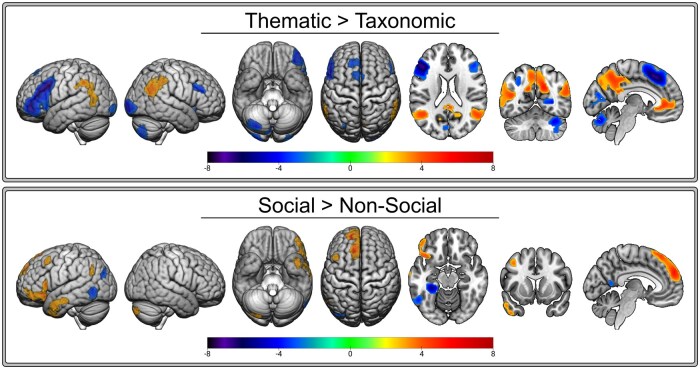
Combined group *Thematic* > *Taxonomic* and *Social* > *Non-Social* results. Warmer colours (yellow to red) indicate greater activation for the first condition (*Thematic*, *Social*) relative to the second condition. Cooler colours (cyan to purple) indicate greater activation for the second condition (*Taxonomic*, *Non-Social*) relative to the first condition.

The results of the whole-brain analyses are presented in Fig. 5 and the coordinate information is provided in Table 4. There were no statistically significant group differences in either thematic-vs-taxonomic or social-vs-non-social contrasts, so the overall results are presented here.

**Table 4.** Coordinate table.

Contrast	Cluster size	Hem	Brain region peak voxel	Brain region highest overlap (%)	MNI coordinates		
					*X*	*Y*	*Z*
Thematic > taxonomic	1345	R	Precuneus	Precuneus (31%)	15	−60	29
	522	R	Anterior cingulate cortex	Left anterior cingulate cortex (30%)	3	40	2
	455	R	Angular gyrus	Angular gyrus (33%)	61	−60	26
	382	L	Middle temporal gyrus	Middle temporal gyrus (54%)	−42	−60	16
Taxonomic > thematic	1488	L	Inferior frontal gyrus (triangularis)	Inferior frontal gyrus (triangularis) (39%)	−48	11	36
	1151	R	Cerebellum (Crus 2)	Right cerebellum (Crus 1) (18%)	9	−82	−44
	639	L	Supplementary motor area	Supplementary motor area (35%)	−3	16	56
	522	R	Fusiform gyrus	Calcarine gyrus (30%)	30	−59	0
	234	R	Inferior frontal gyrus (triangularis)	Inferior frontal gyrus (triangularis) (44%)	52	29	31
	131	R	Inferior frontal gyrus (orbitalis)	Insula (62%)	33	25	−6
	121	L	Middle occipital gyrus	Inferior parietal lobule (39%)	−27	−76	38
Social > non-social	528	L	Superior medial gyrus	Superior medial gyrus (48%)	−6	54	36
	303	L	Inferior frontal gyrus (orbitalis)	Inferior frontal gyrus (Orbitalis) (49%)	−48	25	−13
	182	L	Medial temporal pole	Middle temporal gyrus (58%)	−42	15	−49
	161	R	Cerebellum (Crus 2)	Cerebellum (Crus 2) (53%)	27	−82	−48
	124	L	Angular gyrus	Angular gyrus (84%)	−52	−60	26
	115	L	Middle frontal gyrus	Middle frontal gyrus (91%)	−36	13	50
Non-Social > social	192	L	Inferior temporal gyrus	Inferior temporal gyrus (51%)	−55	−62	−7
	150	R	Precuneus	Precuneus (25%)	12	−50	7
	135	L	Fusiform gyrus	Fusiform gyrus (62%)	−27	−36	−24
	118	L	Middle occipital gyrus	Middle occipital gyrus (97%)	−39	−82	25

Cluster size is determined by the number of 2mm^3^ voxels. % Overlap is the percent overlap between each cluster and the atlas defined regions [based on the Eickhoff-Zilles macro labels from the N27 (MNI space) atlas]. The regions which contained the peak voxels are bolded. MNI coordinates correspond to the voxel with peak activation within each cluster. Voxels were defined as neighbours based on faces touching (NN = 1). These statistical maps are thresholded at a cluster-forming threshold of *P* < .005 (107 voxels) and a family-wise error rate of *P* < .05. Hem, hemisphere; L, left; R, right.

#### Thematic > Taxonomic

When combining across both groups, judging thematic relative to taxonomic relations engaged the default mode network in right precuneus, anterior cingulate cortex, angular gyrus, and dorsomedial prefrontal cortex as well as a posterior portion of left MTG which extended into the angular gyrus cluster. Judging taxonomic relative to thematic relations engaged the semantic control network in left inferior frontal gyrus and to a much lesser extent right inferior frontal gyrus as well as left supplementary motor area, right posterior fusiform, calcarine, and middle occipital gyri. There were no group differences.

#### Social > Non-Social

When combining across both groups, judging social relative to non-social relations engaged largely left hemisphere regions in the superior frontal gyrus (medial part), middle frontal gyrus, the orbitalis portion of the inferior frontal gyrus, medial temporal pole and MTG (covering ventrolateral ATL), angular gyrus, and right cerebellum. Judging non-social relative to social relations engaged a posterior portion of left inferior temporal and fusiform gyri, left MTG, and right precuneus. There were no group differences.

### Region of interest results

**Figure 6.**
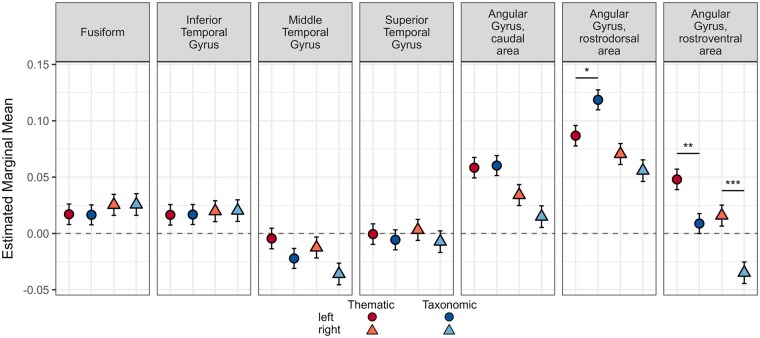
Thematic and taxonomic ROI analysis results. The thematic (red) and taxonomic (blue) extracted model estimates are plotted for each ROI in separate panels. The left hemisphere estimates are indicated with circles and more saturated colours, and the right hemisphere estimates are indicated with triangles and less saturated colours. Significance between conditions is indicated with horizonal bars and asterisks: ^*^P < .05; ^**^P < .01; ^***^P < .001.

**Figure 7.**
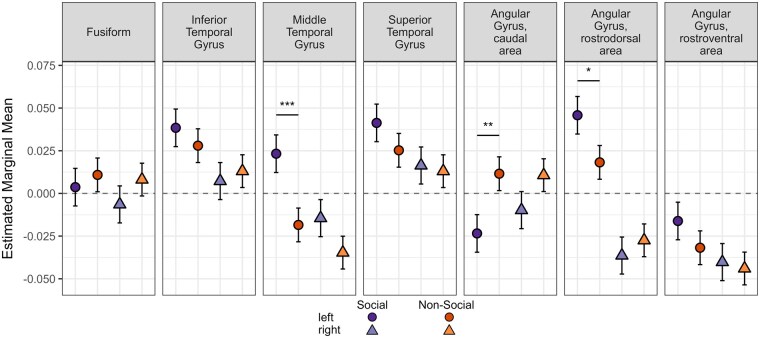
Social and Non-Social ROI analysis results. The social (purple) and non-social (orange) extracted model estimates are plotted for each ROI in separate panels. The left hemisphere estimates are indicated with circles and more saturated colours, and the right hemisphere estimates are indicated with triangles and less saturated colours. Significance between conditions is indicated with horizonal bars and asterisks: ^*^P < .05; ^**^P < .01; ^***^P < .001.

There was no main effect of group in either the taxonomic/­thematic model (*Est.* = 0.007, SE = 0.01, *P* = .41) or the social/non-social model (*Est.* = 0.003, SE = 0.01, *P* = .79). The extracted pairwise contrasts are provided in Supplemental Table 1 (see online supplementary material for a colour version of this table) and visualised in Fig. 6 (taxonomic/thematic model) and Fig. 7 (social/non-social model).

#### Thematic and Taxonomic

There were no differences in engagement of ATL subregions between thematic and taxonomic conditions, suggesting that bilateral ATLs are equally engaged by both types of relations. Consistent with the whole-brain results, condition differences were observed in the rostral portion of angular gyrus. Interestingly, however, the left rostrodorsal area was more active when judging taxonomic relative to thematic relations whereas, bilaterally, the rostroventral area was more active when judging thematic related to taxonomic relations. This pattern of results suggests that, rather than performing a uniform function, the angular gyrus makes varied contributions to semantic cognition.

#### Social and Non-Social

Left MTG was more engaged by social relative to non-social pairs, and although the mean estimate within the social condition was higher in left ITG and STG compared to MTG, these estimates were similar for the non-social condition. Counter to our predictions, the caudal area of left angular gyrus was more active when judging non-social relative to social pairs whereas the left rostrodorsal area (which was more engaged by taxonomic relative to thematic pairs) was more engaged by social relative to non-social pairs. There were no condition differences in the rostroventral area, despite this area having the greatest overlap with the TPJ and the observed cluster in the whole-brain results.

## Discussion

The semantic system represents all manner of conceptual knowledge—objects and events, abstract and concrete, social and non-social concepts. The present study investigated the extent to which distinct semantic regions or hubs represent taxonomic versus thematic relations and social versus non-social relations. This was tested in two groups that ostensibly have different social experiences: autistic and non-autistic adults. We found evidence of group differences in the behavioural but not neural data, and not in the direction predicted by prevailing social theories of autism. Specifically, we found no evidence of a taxonomic or non-social processing bias in autistic adults. Instead, non-autistic adults judged taxonomic relations more accurately and judged social relations less accurately than the autistic adults did. Although caution should be taken in interpreting a null finding in a relatively small sample, this suggests that (mis)understanding of social concepts does not drive differences in social interaction. The semantic and social deficit accounts of autism—as they are typically articulated—may require revision. A more fruitful direction for understanding differences in the social experiences and knowledge between autistic and non-autistic people may be to explore social dynamics at the interactional level, in line with the claims made by the double empathy account (Milton 2012). Given the lack of group differences in the fMRI data, the following sections discuss the results across all participants.

### Thematic versus Taxonomic

In whole-brain analyses, bilateral angular gyrus was more active when processing thematic compared to taxonomic relations, but there was no complementary specialisation for taxonomic relations in ATL. This provides partial support to a dual-hub account of semantic cognition and is consistent with recent research reporting a similar pattern of asymmetric specialisation (Zhang et al. 2023, Zhang et al. 2024), although some studies do report both ATL and angular gyrus specialisation (Xu et al. 2018, Thye et al. 2021). In ROI analyses, all ATL subregions were approximately equally active for taxonomic and thematic relations, consistent with the whole-brain analyses and prior work showing ATL engagement across semantic tasks, presentation modalities, and for a range of conceptual categories (Hoffman et al. 2015, Lambon Ralph et al. 2017). The ROI analyses paint a complex picture of the specialisation within angular gyrus: the left rostrodorsal area was more active when processing taxonomic relative to thematic relations whereas, bilaterally, the rostroventral area was more active when processing thematic relative to taxonomic relations. It was the rostroventral area too where overlap was observed between the social (relative to non-social) and thematic (relative to taxonomic) whole-brain results. This pattern suggests graded functioning in angular gyrus such that subregions represent different types of semantic relations, with the rostroventral area showing the strongest thematic effect in both the whole-brain and ROI analyses. Variations in the representation of semantic knowledge across the angular gyrus may explain inconsistent findings regarding thematic versus taxonomic specialisation in prior work.

Whole-brain analyses also suggested a network dissociation such that thematic relations engaged the broader default mode network whereas taxonomic relations engaged the semantic control network, particularly IFG. This cannot be attributed to differences in task-demand given that the conditions were well-matched, accuracy was high in both conditions, and there were no condition differences in accuracy or reaction time. Although the non-autistic group judged the taxonomic pairs more accurately than the autistic group did, there were no group differences in the neural data. This indicates that even if one group found the pairs easier to judge than the other, taxonomic pairs were not generally harder to judge than thematic pairs were, and the underlying networks were the same for both groups. This pattern is also surprising given deactivation of the DMN across attention demanding, externally oriented semantic tasks (Shao et al. 2024). However, it is possible that DMN regions play an active role in semantic cognition—indeed, they are observed in large meta-analyses of linguistic stimuli (Binder et al. 2009) and representational similarity analyses have associated word embeddings from language models with activity patterns within these regions (Fernandino et al. 2022, Graves et al. 2023). DMN regions, including the angular gyrus, may be particularly important for these associative semantic representations which our thematic relations are likely capturing. Taxonomic relations, in contrast, are more specific and may engage semantic control regions for selecting the relevant semantic features for identifying the taxonomic relationship (Jefferies et al. 2020). This distinction may also be helpful for understanding taxonomic-thematic dissociations within the angular gyrus. While the ventral parts of this region fall within the DMN, the dorsal part is linked to cognitive control, including in semantic tasks (Humphreys and Lambon Ralph 2015). There was also notable engagement of visual areas when judging taxonomic relative to thematic relations. The ventral ATL receives inputs from the ventral visual stream or ‘what’ pathway for processing information about objects and their features (Mishkin et al. 1983). Connections to and engagement of this pathway is expected to be greater for taxonomic knowledge given that visual similarity is often tightly linked to taxonomic similarity. As a result, visual features are activated when processing taxonomic relative to thematic pairs. We did not observe corresponding activation along the dorsal or ‘where/how’ pathway which might be more involved in processing thematic relations.

### Social versus Non-Social

In whole-brain analyses, the differences between social versus non-social relations were primarily in the left middle temporal portion of ventrolateral ATL, the rostroventral area of angular gyrus, inferior frontal gyrus, and other frontal regions. This pattern overlaps with the social brain network regions identified in a prior meta-analysis (Diveica et al. 2021). The ventrolateral ATL region in particular has been observed in other studies of social semantics and social cognition more broadly (Binney et al. 2016, Balgova et al. 2022). Although part of the graded ATL semantic hub region, the anterior MTG is not the portion of the hub most consistently implicated as being transmodal—the ventral surface of ATL, centred on anterior fusiform, is thought to be transmodal, representing all conceptual categories across modalities (Lambon Ralph et al. 2017). Consistent with this, we did not observe ventral ATL activation differences in any of the contrasts despite using a multi-echo sequence optimised for maximizing signal in the ventral ATL. That is, the transmodal portion of the hub is equally engaged across conditions, which was confirmed with ROI analyses examining condition differences within ATL subregions. We also did not observe engagement of STG for social relative to non-social words, despite prior work which reported social effects in STG rather than MTG (Zahn et al. 2007, Mellem et al. 2016, Catricalà et al. 2020).

There are a few possible interpretations for greater anterior MTG engagement for social concepts. The first is that this subregion is specialised for social semantics. Although this explanation is consistent with the present results, it runs counter to a body of research reporting ATL engagement in response to varied non-social, semantic stimuli (Lambon Ralph et al. 2017). The second interpretation is that the anterior MTG interfaces with the DMN and the response to social concepts reflects a greater need to engage in context-driven or self-referential processing that this network supports (Thye et al. 2024a, 2024b). This claim is supported by evidence of particularly strong structural and functional connectivity between anterior MTG and the DMN (Simony et al. 2016, Sasaki et al. 2023).

No additional DMN engagement was observed for social relations (relative to non-social), but there was broad DMN engagement for thematic relations (relative to taxonomic). Both social and non-social concepts were abstract in this study and the increased control demands of processing abstract concepts (Rice et al. 2018) may have reduced DMN activity. This interpretation is supported by the abstract versus concrete contrast for the present study, shown in Supplemental Figure 1 (see online supplementary material for a colour version of this figure) and Supplemental Table 2 (see online supplementary material for a colour version of this table), which found greater activation in DMN regions for concrete concepts. However, this pattern is surprising given that DMN regions are often included in definitions of the social cognition network (Adolphs 2001, 2009).

In the whole-brain analyses, an overlapping portion of rostroventral angular gyrus was engaged for thematic and social relations relative to their contrasted conditions. Compared to the other subregions, this portion of the angular gyrus has the greatest overlap with the TPJ, a region consistently regarded as contributing to social cognition. Engagement of this region for social relative to non-social concepts is consistent with the role of this region in social processing, but overlap with the thematic results suggest that a domain-general mechanism underlies both processes.

However, this pattern was not confirmed in the ROI analyses. This is likely because the rostroventral ROIs were relatively large with respect to the small overlap cluster that whole-brain results identified within this region. Future research could define a ‘social’ angular gyrus functional ROI within participants using well-established social cognition localizer tasks and then examine engagement within this region to social concepts and thematic relations. Approaches like multi voxel pattern analysis may also be useful in localizing the portions of angular gyrus that are responsive to thematic and social content.

The angular gyrus has been implicated in diverse functions and networks: semantic cognition (possibly specialised for thematic/event semantics), social cognition, autobiographical memory, attention, and as part of the DMN (Rockland and Graves 2023, Seghier 2023, 2024). Therefore, domain-specific accounts of the role of this region in cognition are not viable; rather, an adequate account would need to explain why this region is particularly important for thematic semantics *and* social cognition *and* autobiographical memory *and* attention *and*, especially, its role within the DMN. Accounts implicating this region in the construction of situation models—mental representations of a situation of series of events—represent a promising step in this direction (Yarkoni et al. 2008, Lee et al. 2020, Humphreys and Tibon 2023). Situation models are particularly relevant to thematic relations, which occur in specific spatiotemporal contexts, and to social concepts, which generally refer to interactions taking place in well-defined social situations.

## Conclusion

There was minimal evidence of differences in semantic processing between autistic and non-autistic adults for the conceptual categories included in the present study. Taxonomic relations engaged the semantic control system to a greater extent than thematic relations did, which instead engaged the rostroventral area of angular gyrus and other portions of the default mode network. The same portion of the rostroventral area of left angular gyrus supported the processing of thematic and social relations, but the broader network underlying their representation and retrieval differed, likely due, in part, to differences in abstractness between the conditions. The ATLs were approximately equally engaged by thematic and taxonomic relations, but MTG was more active for social relative to non-social concepts. The results are broadly consistent with a dual-hub account of semantic cognition, but suggest that angular gyrus represents semantic knowledge in a graded fashion and a portion of the rostroventral area of left angular gyrus—which overlaps with TPJ—may be particularly sensitive to context-driven processing that is relevant for both thematic and social relations.

## Supplementary Material

nsaf079_Supplementary_Data

## Data Availability

The data, code, and materials underlying this article are available at https://osf.io/5xasw.
