# Supplementary material for: Neural correlates of social and thematic semantics in autistic and non-autistic adults
Source: Soc Cogn Affect Neurosci. 2025 Jul 24;20(1):nsaf079. doi: 10.1093/scan/nsaf079 (PMC12526934; doi:10.1093/scan/nsaf079)
Supplement: nsaf079_Supplementary_Data [file nsaf079_supplementary_data.zip › scan-25-091-File016.docx]

**Supplementary Information**

**Stimulus Selection**

Candidate stimuli were derived from a set of publicly available word norms of taxonomic (*dog* – *bear*) and thematic relations (*dog* - *leash*) (Landrigan & Mirman, 2016) and the Glasgow norms study, which includes normative psycholinguistic ratings for over 5000 individual words (Scott et al., 2018). The latter list was filtered to remove words with high concreteness (> 5) and imageability (> 5) ratings in order to identify abstract words as norming targets. Additional target words were added from a study reporting social desirability ratings on over 500 words (Hampson et al., 1987). To obtain ratings of socialness, social word ratings were generated from a previous norming study conducted with 68 participants from the University of Alabama at Birmingham. A randomly selected subset of 688 words were included in the norming study. Each participant rated half of the words resulting in 34 socialness ratings for each of the 688 unique words. Participants were instructed that a word is social if it describes inter-personal behaviours, motivations, intentions, or characteristics and were asked to rate how social each presented word was on a scale from 1 (not social) to 5 (very social). This definition aligns with how socialness has typically been characterised in studies of social cognition and behaviour; however it is important to note that there is no current consensus definition of “socialness” and recent work has argued for a more inclusive definition than the one adopted here (Diveica et al., 2023).

Relatedness between candidate word pairs was calculated using cosine similarity based on pre-trained semantic vectors using word2vec and by obtaining relatedness judgments via Prolific from participants who did not take part in the fMRI experiment. Only word pairs with high cosine similarity and that were consistently judged to be related were retained. Filler trials of unrelated word pairs were constructed using a mix of concrete, abstract non-social, and abstract social words that had low relatedness based on cosine similarity and participant ratings.

On average, the critical word pairs were rated by 32 participants (range = 13-71) and were reliably judged as related (mean accuracy = 90.56%; range = 76%-100%) within the targeted stimulus presentation window of 2.5s (mean RT = 1.35s; range = 1.01-1.70s). Only highly related taxonomic and thematic pairs were selected from the prior norming study, and there was a dissociation in the ratings such that taxonomic pairs were weakly thematically related and vice versa.

**Supplemental Table 1**

*Pairwise ROI comparisons*

|  |  | *Thematic > Taxonomic* | | | *Social > Non-Social* | | |
| --- | --- | --- | --- | --- | --- | --- | --- |
| **Region** | **Hem** | **Estimate** | **SE** | ***p*** | **Estimate** | **SE** | ***p*** |
| Fusiform | L | 0.001 | 0.012 | 0.968 | -0.007 | 0.012 | 0.561 |
| Fusiform | R | -0.000 | 0.012 | 0.980 | -0.015 | 0.012 | 0.239 |
| ITG | L | -0.000 | 0.012 | 0.984 | 0.010 | 0.012 | 0.397 |
| ITG | R | -0.001 | 0.012 | 0.967 | -0.006 | 0.012 | 0.638 |
| MTG | L | 0.018 | 0.012 | 0.152 | 0.042 | 0.012 | 0.001 |
| MTG | R | 0.023 | 0.012 | 0.057 | 0.020 | 0.012 | 0.103 |
| STG | L | 0.005 | 0.012 | 0.411 | 0.016 | 0.012 | 0.194 |
| STG | R | 0.010 | 0.012 | 0.398 | 0.003 | 0.012 | 0.787 |
| AG, rostrodorsal | L | -0.032 | 0.012 | 0.010 | 0.028 | 0.012 | 0.026 |
| AG, rostrodorsal | R | 0.015 | 0.012 | 0.232 | -0.009 | 0.012 | 0.470 |
| AG, rostroventral | L | 0.039 | 0.012 | 0.002 | 0.016 | 0.012 | 0.205 |
| AG, rostroventral | R | 0.051 | 0.012 | <.0001 | 0.004 | 0.012 | 0.761 |
| AG, caudal | L | -0.002 | 0.012 | 0.879 | -0.035 | 0.012 | 0.005 |
| AG, caudal | R | 0.019 | 0.012 | 0.120 | -0.020 | 0.012 | 0.098 |

*Note.* Hem, hemisphere; L, left; R, right; SE, standard error; ITG, inferior temporal gyrus; MTG, middle temporal gyrus; STG, superior temporal gyrus; AG, angular gyrus.

**Abstract > Concrete Analysis**

To estimate neural response to the processing of abstract versus concrete relations, the social and non-social (*Abstract*) were contrasted with the taxonomic and thematic (*Concrete*) conditions across both groups (**Supplemental Figure 1**). Coordinate information is provided in **Supplemental Table 2**. As note, the *Abstract* and *Concrete* conditions were not matched on word frequency and semantic diversity (higher for *Concrete* pairs) or number of letters (greater for *Abstract* pairs). As expected, the conditions differed in concreteness and imageability, which were higher for the *Concrete* pairs.

**
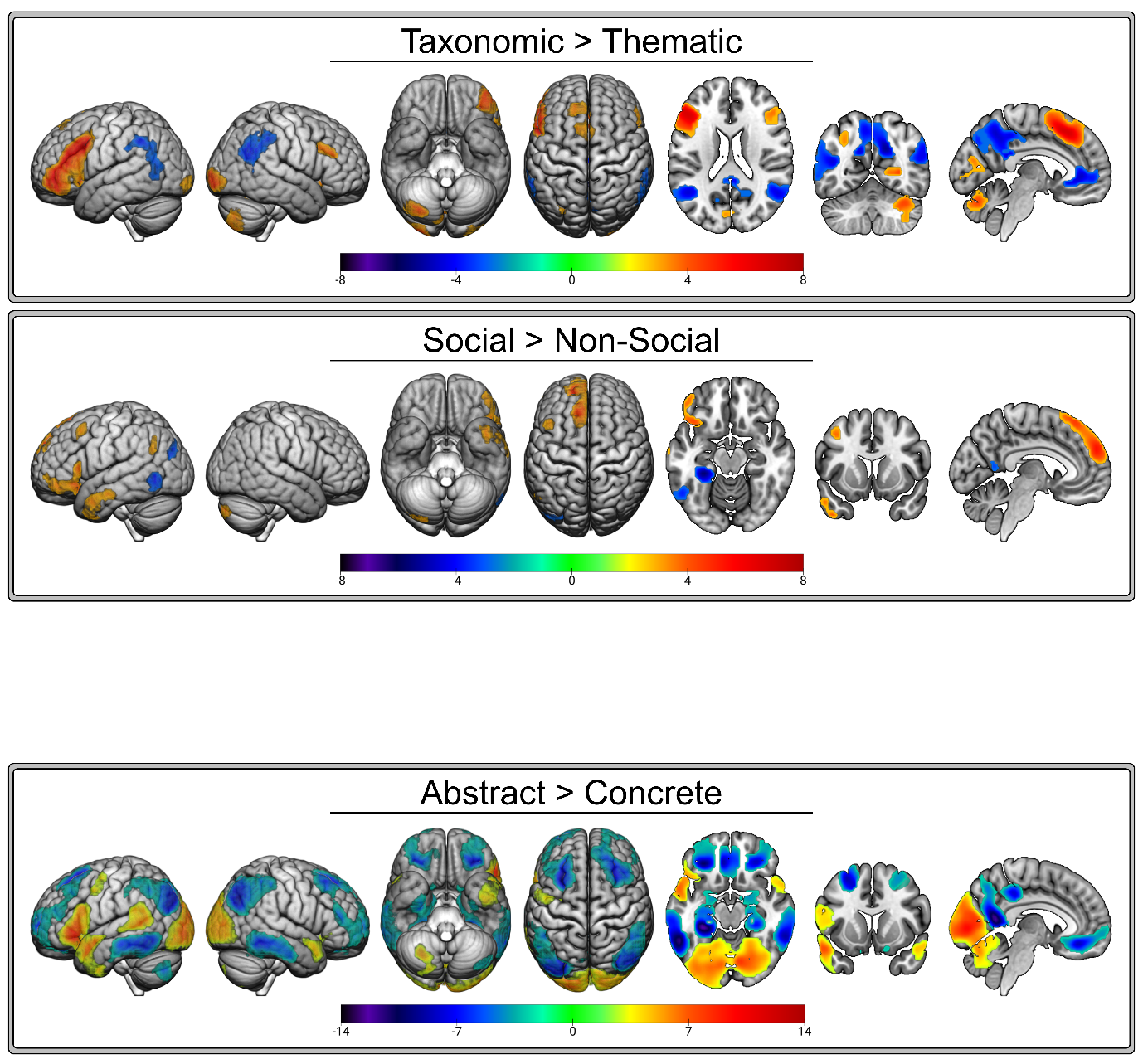
**

**Supplemental Figure 1.** Combined group *Abstract > Concrete* results. Warmer colours (yellow to red) indicate greater activation for *Abstract* relative to *Concrete* relations. Cooler colours (cyan to purple) indicate greater activation for the *Concrete* relative to *Abstract* relations.

**Supplemental Table 2**

*Abstract > Concrete Coordinate Table*

| **Contrast** | **Cluster Size** | **Hem** | **Brain Region**  **Peak Voxel** | **Brain Region**  **Highest Overlap [%]** | **MNI Coordinates** | | |
| --- | --- | --- | --- | --- | --- | --- | --- |
|  |  |  |  |  | **X** | **Y** | **Z** |
| *Abstract > Concrete* | 6087 | R | Lingual Gyrus | Lingual Gyrus [8%] | 15 | -83 | -5 |
|  | 1205 | L | Inferior Frontal Gyrus (Triangularis) | Inferior Frontal Gyrus (Triangularis) [21%] | -48 | 28 | -2 |
|  | 553 | L | Middle Temporal Gyrus | Middle Temporal Gyrus [63%] | -52 | -43 | 1 |
|  | 282 | R | Temporal Pole | Insula [31%] | 48 | 13 | -24 |
|  | 241 | L | Precentral Gyrus | Precentral Gyrus [84%] | -48 | -2 | 46 |
|  | 192 | R | Superior Temporal Gyrus | Superior Temporal Gyrus [44%] | 42 | -40 | 4 |
| *Concrete > Abstract* | 3899 | L | Middle Frontal Gyrus | Middle Frontal Gyrus [19%] | -24 | 16 | 60 |
|  | 1202 | R | Inferior Temporal Gyrus | Inferior Temporal Gyrus [34%] | 61 | -46 | -21 |
|  | 1078 | L | Middle Occipital Gyrus | Inferior Parietal Lobule [32%] | -33 | -85 | 35 |
|  | 1050 | R | Angular Gyrus | Angular Gyrus [37%] | 48 | -73 | 32 |
|  | 925 | L | Precuneus | Precuneus [31%] | -6 | -56 | 10 |
|  | 697 | L | Fusiform Gyrus | Fusiform Gyrus [35%] | -27 | -36 | -24 |
|  | 612 | L | Inferior Temporal Gyrus | Inferior Temporal Gyrus [59%] | -55 | -52 | -17 |
|  | 178 | L | Cerebellum (Crus 2) | Cerebellum (Crus 2) [73%] | -39 | -72 | -54 |

*Note.* Hem, Hemisphere; L, Left; R, Right. Cluster size is determined by the number of 2mm^3^ voxels. % Overlap is the percent overlap between each cluster and the atlas defined regions (based on the Eickhoff-Zilles macro labels from the N27 (MNI space) atlas). The regions which contained the peak voxels are bolded. MNI coordinates correspond to the voxel with peak activation within each cluster. Voxels were defined as neighbours based on faces touching (NN=1). These statistical maps are thresholded at a cluster-forming threshold of *p* < .05 (107 voxels) and a family-wise error rate of *p* < .005.

**References**

Diveica, V., Pexman, P. M., & Binney, R. J. (2023). Quantifying social semantics: An inclusive definition of socialness and ratings for 8388 English words. *Behavior Research Methods*, *55*(2), 461–473. https://doi.org/10.3758/s13428-022-01810-x

Hampson, S. E., Goldberg, L. R., & John, O. P. (1987). Category-breadth and social-desirability values for 573 personality terms. *European Journal of Personality*, *1*(4), 241–258. https://doi.org/10.1002/per.2410010405

Landrigan, J.-F., & Mirman, D. (2016). Taxonomic and Thematic Relatedness Ratings for 659 Word Pairs. *Journal of Open Psychology Data*, *4*(1), 2. https://doi.org/10.5334/jopd.24

Scott, G. G., Keitel, A., Becirspahic, M., Yao, B., & Sereno, S. C. (2018). The Glasgow Norms: Ratings of 5,500 words on nine scales. *Behavior Research Methods*, 1–13. https://doi.org/10.3758/s13428-018-1099-3
